# Transcriptional control of a collagen deposition and adhesion process that promotes lung adenocarcinoma growth and metastasis

**DOI:** 10.1172/jci.insight.153948

**Published:** 2022-01-11

**Authors:** Xiaochao Tan, Priyam Banerjee, Xin Liu, Jiang Yu, Sieun Lee, Young-Ho Ahn, Chad J. Creighton, Jonathan M. Kurie

**Affiliations:** 1 Department of Thoracic/Head and Neck Medical Oncology, The University of Texas MD Anderson Cancer Center, Houston, Texas, USA.; 2 Department of Molecular Medicine, College of Medicine, Ewha Womans University, Seoul, South Korea.; 3 Department of Medicine and Dan L. Duncan Cancer Center, Baylor College of Medicine, Houston, Texas, USA.; 4 Department of Bioinformatics and Computational Biology, The University of Texas MD Anderson Cancer Center, Houston, Texas, USA.

**Keywords:** Oncology, Cell migration/adhesion, Collagens, Integrins

## Abstract

A fibrotic stroma accumulates in advanced cancers, and invasive cancer cells migrate along collagen fibers that facilitate dissemination from the primary tumor. However, the ways in which tumor cells govern these processes remain unclear. Here, we report that the epithelial-mesenchymal transition–activating transcription factor ZEB1 increased type I collagen (Col1) secretion and enhanced tumor cell adherence to Col1. Mechanistically, ZEB1 increased the levels of α_1_β_1_ integrin (encoded by *Itga1* and *Itgb1*) by inhibiting PP2A activity, which reduced nuclear accumulation of HDAC4 and, thereby, derepressed *Itga1* gene transcription. In parallel, ZEB1 relieved the miRNA-148a-mediated silencing of Itga1. High levels of Itga1 enhanced tumor cell adherence to Col1 and were essential for Col1-induced tumor growth and metastasis. Furthermore, ZEB1 enhanced Col1 secretion by increasing the expression of a kinesin protein that facilitated transport and secretion of Col1-containing vesicles. Our findings elucidate a transcriptional mechanism by which lung adenocarcinoma cells coordinate a collagen deposition and adhesion process that facilitates tumor progression.

## Graphical abstract

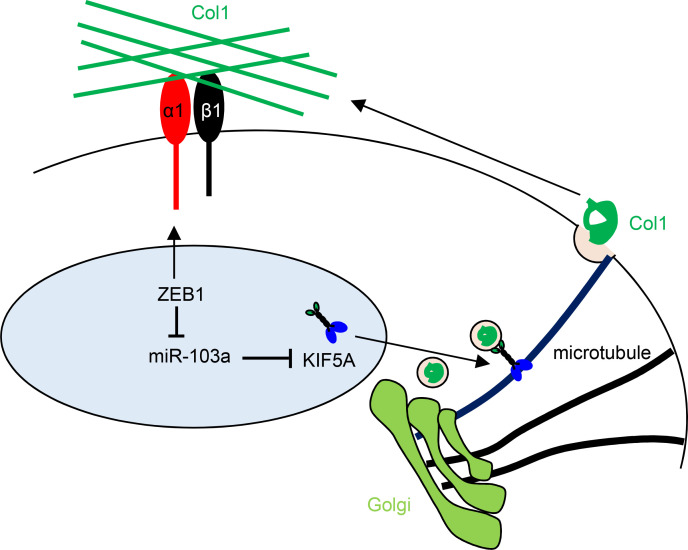

## Introduction

Metastasis remains the leading cause of cancer-related death (1). In certain types of epithelial tumors, metastasis requires cancer cells to switch between epithelial and mesenchymal states (2). This reversible switch is regulated by mutual antagonism between transcription factors that promote epithelial-mesenchymal transition (EMT; e.g., ZEB, SNAIL, and TWIST family members) and miRNAs that target EMT-activating transcription factors (e.g., miR-200 family members and miR-34a) (2). Although the role of EMT in metastasis varies depending on tumor type (3–5), it is generally accepted that EMT and the reverse process play key roles in the metastatic cascade (6).

A dense collagenous matrix accumulates in advanced malignancies and facilitates cancer cell survival and dissemination (7, 8). Collagen molecules generate triple helical structures that are secreted into the extracellular space where they form cross-linked, fibrillar bands that maintain the integrity of the extracellular matrix (9). Collagen-enhanced stromal stiffness activates integrins that increase tumor cell motility and dissemination, and the invasive tumor cells in turn remodel collagen fibers and invade along the realigned collagen fibers (10, 11). The types of collagen fibers that tumor cells encounter during the initial stages of metastasis include network-forming collagens (e.g., type IV collagen) that line basement membranes and fibrillar collagens (e.g., types I, II, and III) within tissue stroma (12). DDR1, DDR2, and certain members of the integrin family function as collagen receptors (13). The integrin family consists of 18 α and 8 β subunits, which form 24 heterodimeric receptors, 4 of which (α_1_β_1_, α_2_β_1_, α_10_β_1_, and α_11_β_1_) are bona fide collagen receptors (14). Type I collagen (Col1), a major component of the tumor stroma, has been linked to tumor growth and metastasis (15–18). Although Col1 was initially thought to be mainly secreted by fibroblasts (19), growing evidence shows that tumor cells also contribute to collagen deposition (17). However, the ways in which tumor cells govern these processes remain unclear.

In this study, we addressed these questions using tumor cell lines isolated from mice that develop lung adenocarcinoma from expression of K-ras^G12D^ and p53^R172H^. These cell lines (hereafter termed KP cells) exhibit variable metastatic properties. Highly metastatic KP cells exhibit mesenchymal features driven by the EMT-activating transcription factor ZEB1 but revert to an epithelial state and lose their metastatic activity following ectopic expression of the miR-200b/c/429 cluster (20). Poorly metastatic KP cells exhibit epithelial features but undergo mesenchymal differentiation and gain metastatic activity following ectopic expression of ZEB1 (20, 21). The findings presented here support a model in which ZEB1 activates a transcriptional program that coordinates collagen deposition and attachment to facilitate tumor progression.

## Results

### ZEB1 increases tumor cell adherence to collagen through α_1_β_1_ integrin.

**Figure 1:**
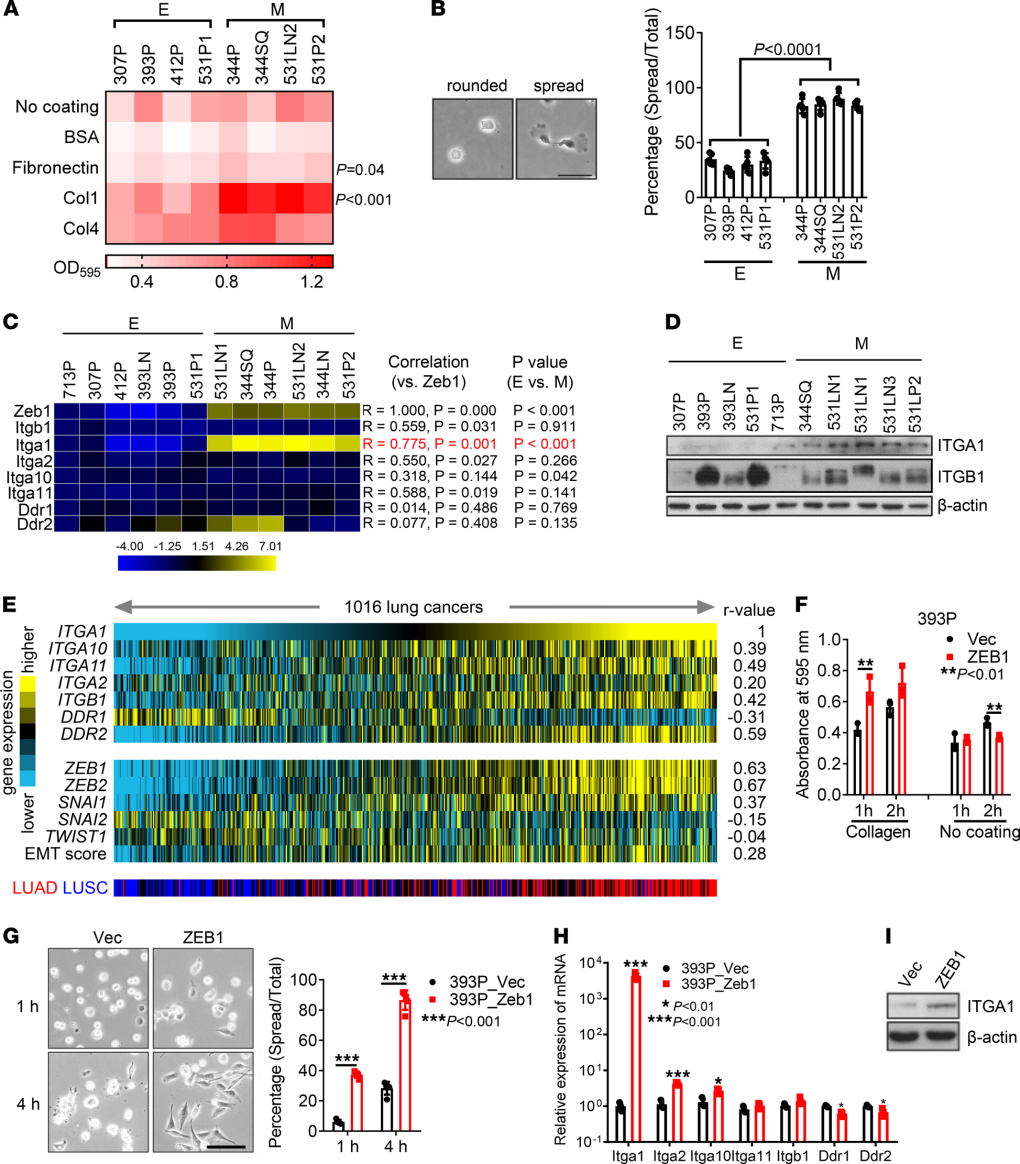
Epithelial-mesenchymal transition increases tumor cell adherence to collagen through α_1_β_1_ integrin. (**A**) Cell adherence was quantified 1 hour after the indicated KP cells were seeded on wells coated with collagen 1 (Col1), collagen IV (Col4), fibronectin, BSA, or nothing, and OD_595_ values are shown in the heatmap. E, epithelial; M, mesenchymal. *n* = 4. (**B**) Cell spreading assay on Col1. Cells were plated in Col1-coated plates for 1 hour, and percentages of spread cells were quantified. Scale bar: 50 μm. (**C**) Quantitative reverse transcription PCR analysis of the mRNA levels of ZEB1 and Col1 receptors in KP cell lines (Pearson correlation). (**D**) Western blot analysis of Itga1 and Itgb1 in KP cell lines classified as epithelial or mesenchymal. β-Actin was used as a loading control. (**E**) Heatmap illustration of gene expression levels in lung adenocarcinoma and squamous cell carcinoma data sets (*n* = 1016 tumors) in The Cancer Genome Atlas. An epithelial-mesenchymal transition score was correlated with the levels of collagen receptors using the Pearson coefficient (*r* value). (**F**) Cell adherence was quantified 1 hour or 2 hours after the indicated cells were seeded on uncoated wells or on wells coated with Col1. *n* = 4. **P < 0.01. (**G**) Cell spreading assay on Col1. Percentages of spread cells were quantified. Scale bar: 100 μm. ***P < 0.001. (**H**) Quantitative reverse transcription PCR analysis of Col1 receptors. (**I**) Western blot analysis of Itga1 in 393P_Vec and 393P_ZEB1 cells. Data are shown as the mean ± SEM from a single experiment incorporating biological replicate samples (*n* = 3, unless otherwise indicated) and are representative of at least 2 independent experiments. *P < 0.01; ***P < 0.001. Two-tailed Student’s *t* test.

To determine whether tumor cell adherence to Col1 is regulated during EMT, we initially quantified cell attachment to collagen- or fibronectin-coated wells using a panel of KP cell lines classified previously as mesenchymal or epithelial (Supplemental Table 1; supplemental material available online with this article; https://doi.org/10.1172/jci.insight.153948DS1) (20). When quantified 1 hour after seeding, attachment to Col1 was correlated positively with mesenchymal classification, whereas attachment did not differ on collagen IV–coated wells or in the absence of collagen (Figure 1A and Supplemental Figure 1A). Furthermore, cell spreading on Col1-coated wells occurred in a greater percentage of mesenchymal KP cells compared with epithelial KP cells (Figure 1B).

To determine whether the increased attachment to collagen results from an EMT-induced change in the expression of one or more of the collagen receptors, we initially quantified the expression of collagen-binding integrins and DDR family members in the KP cell line panel. Expression levels of Itga1, but not other α or β integrins or DDRs, were correlated positively with mesenchymal classification (Figure 1, C and D, and Supplemental Figure 1B). In a compendium of human lung adenocarcinoma cohorts (22), multiple collagen-binding receptors, including Itga1, were positively correlated with the presence of an EMT-related gene expression signature (Figure 1E). In The Cancer Genome Atlas, ZEB1 mRNA levels are positively correlated with ITGA1 mRNA levels in multiple cancer types (Supplemental Figure 1C). EMT induction in epithelial KP cells (393P) by ectopic ZEB1 expression (20, 21) led to enhanced attachment to collagen and spreading on collagen (Figure 1, F and G). Furthermore, ectopic expression of ZEB1 strongly increased the mRNA and protein levels of Itga1 in both murine and human epithelial lung cancer cells (Figure 1, H and I, and Supplemental Figure 1, D and E).

**Figure 2:**
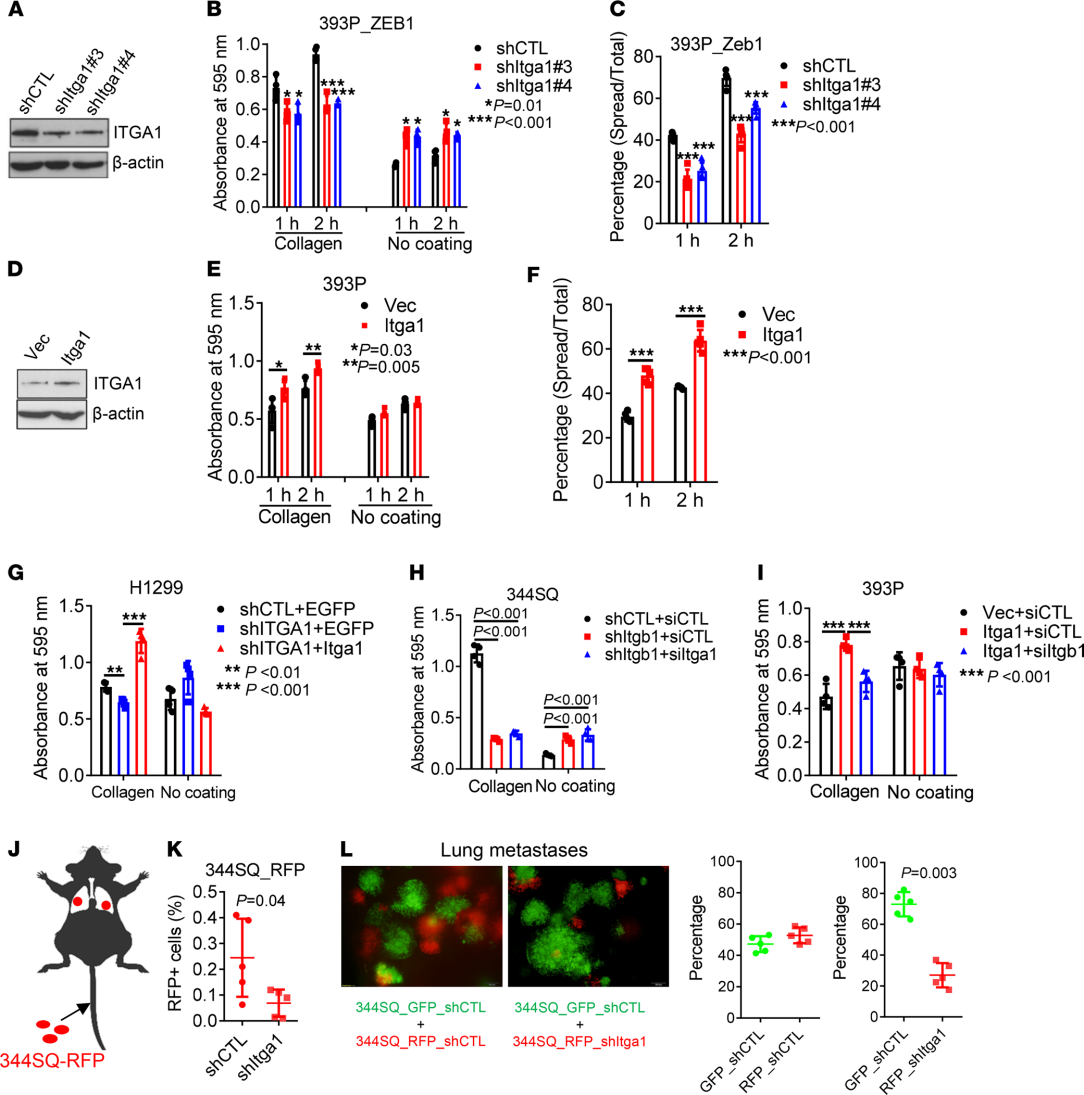
ZEB1 increases tumor cell adherence to Col1 through α_1_β_1_ integrin. (**A**) Western blot analysis of Itga1 in 393P_ZEB1 cells transfected with Itga1 shRNAs (shItga1) or control shRNA (shCTL). (**B**) Cell adhesion to Col1 or plastic (no coating) at indicated time points. *n* = 4. *P = 0.01; ***P < 0.001. (**C**) Percentages of spread cells on collagen at indicated time points. ***P < 0.001. (**D**) Western blot analysis of Itga1 in 393P cells transfected with Itga1 expression vector or control vector (Vec). (**E**) Cell adhesion to Col1 or plastic at indicated time points. *n* = 4. *P = 0.03; **P = 0.005. (**F**) Percentages of spread cells on Col1 at indicated time points. ***P < 0.001. (**G–I**) Cell adhesion to collagen or plastic. H1299 cells transfected with ITGA1 shRNA or control shRNA (shCTL) and Itga1 expression vector or empty vector (Vec) (**G**), 344SQ cells transfected with shRNA against Itgb1 or control shRNA and siRNA against Itga1 or control siRNA (siCTL; **H**), and 393P cells transfected with Iga1 expression vector or control vector and siRNA against Itgb1 or siCTL (**I**) are shown. *n* = 4. **P < 0.01; ***P < 0.001. (**J**) Schema showing tail vein injection of red fluorescent protein–labeled (RFP-labeled) 344SQ cells into mice. (**K**) Percentage of RFP-positive cells in mouse lungs at 4 hours after injection. (**L**) Green fluorescent protein–labeled (GFP-labeled) and RFP-labeled 344SQ transfectants were coinjected into mice and the resulting micrometastases were imaged and quantified. Data are shown as the mean ± SEM from a single experiment incorporating biological replicate samples (*n* = 3, unless otherwise indicated) and are representative of at least 2 independent experiments. Two-tailed Student’s *t* test for 2-group comparisons; 1-way ANOVA test for multiple comparisons.

To determine whether Itga1 expression is required for attachment on Col1, we depleted Itga1 in mesenchymal lung cancer cells (393P_ZEB1, 344SQ, and H1299) and found that Itga1 deficiency led to reduced attachment to Col1 and cell spreading on Col1-coated wells (Figure 2, A–C, and Supplemental Figure 2, A–F). Ectopic expression of Itga1 in 393P or Itga1-depleted H1299 cells restored attachment to Col1 (Figure 2, D–G, and Supplemental Figure 2G). Consistent with a previous report (23), Itga1 colocalized and formed a complex with Itgb1 (Supplemental Figure 2, H and I). Depletion of Itgb1 phenocopied the effects of Itga1 depletion and abrogated the effects of ectopic Itga1 expression on attachment (Figure 2, H and I, and Supplemental Figure 2, J and K). When intravenously injected into mice, Itga1-deficient 344SQ cells showed less lung retention (Figure 2, J and K) and generated fewer micrometastases than did Itga1-replete cells (Figure 2L and Supplemental Figure 2L). Taken together, these findings suggest that ZEB1 increases tumor cell affinity to Col1 by increasing Itga1 expression.

### Itga1 mediates collagen-induced tumor growth and metastasis.

**Figure 3:**
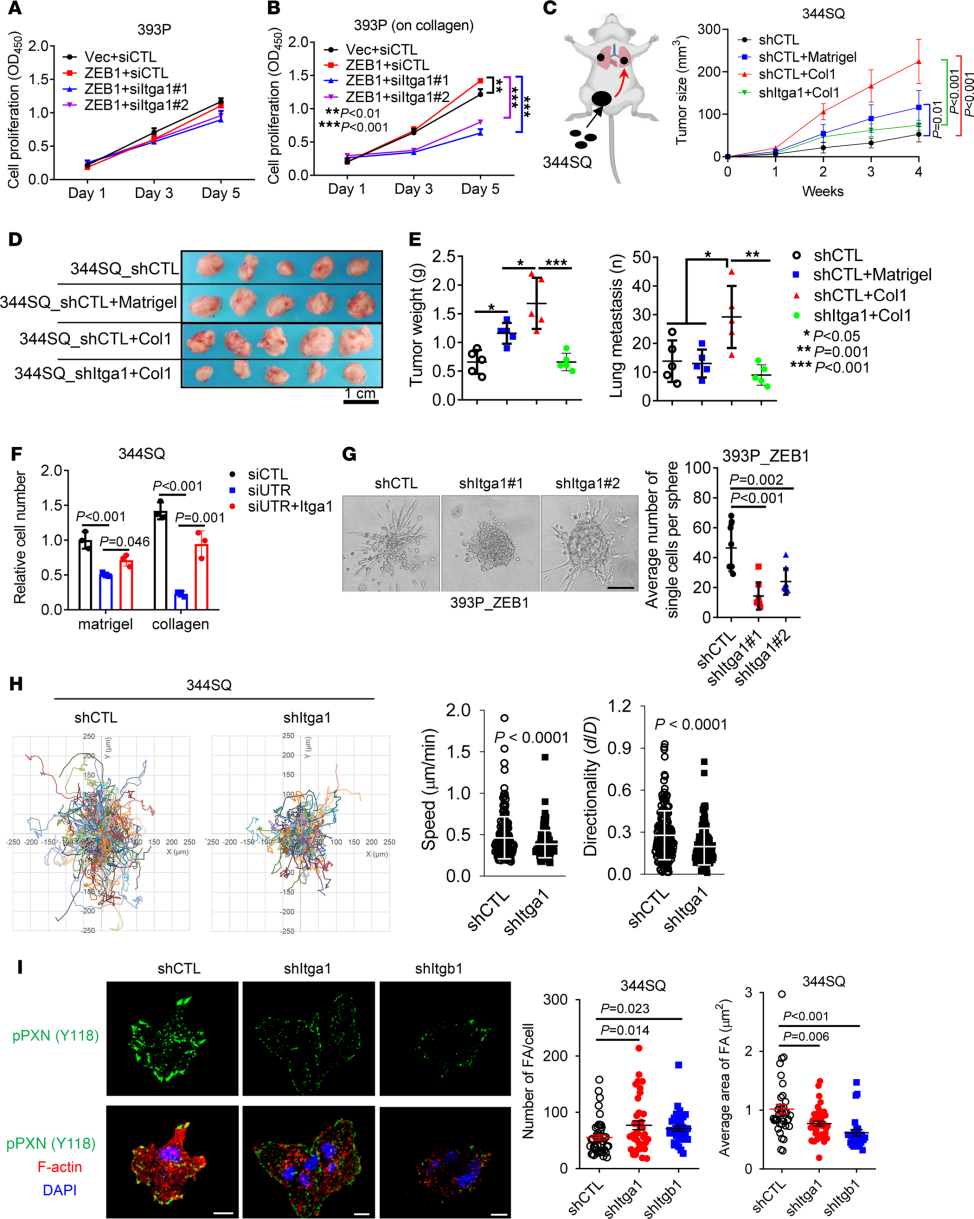
Itga1 mediates Col1-induced cell growth and metastasis. (**A** and **B**) Cell proliferation (**A**) on plastic or (**B**) on Col1. *n* = 5. **P < 0.01; ***P < 0.001. (**C–E**) 344SQ transfectants were coinjected with 5% Matrigel, 5% Col1, or phosphate-buffered saline, and (**C**) tumor volumes were measured weekly. (**D** and **E**) Primary flank tumor weight and number of lung metastases in each mouse were measured and quantified at necropsy. *n* = 5. Scale bar: 1 cm. *P < 0.05; **P = 0.001; ***P < 0.001. (**F**) 344SQ cells were transfected with siRNA targeting the 3′ untranslated region of Itga1 mRNA (siUTR) or control siRNA (siCTL) and Itga1 expression vector or empty vector, and the transfectants were subjected to Boyden chamber Matrigel and Col1 invasion assays. Invaded cells were quantified. (**G**) Bright-field micrograph of a 393P_ZEB1 spheroid in Col1 showing cells invading singly or collectively. Invading single cells per sphere were quantified (graph). Scale bar: 100 μm. (**H**) Time-lapse tracing of movements of 344SQ cell transfectants on Col1. Movements of individual cells over 6 hours are indicated by colored lines. Bar graphs show the quantification of cell speed and directionality. (**I**) Confocal micrographs of focal adhesions (tyrosine 118 phosphorylated paxillin (pPXN [Y118]), green) and nuclei (DAPI, blue) in 344SQ cell transfectants. Scale bar: 10 μm. The scatter plots show the number of focal adhesions (FA) and area of FA in each cell (dots). Data are shown as the mean ± SEM from a single experiment incorporating biological replicate samples (*n* = 3, unless otherwise indicated) and are representative of at least 2 independent experiments. Two-tailed Student’s *t* test for 2-group comparisons; 1-way ANOVA test for multiple comparisons.

Cancer cells exposed to collagen show increased proliferative and invasive activity (24). We postulated that Itga1-mediated collagen adherence is required for ZEB1-driven lung cancer progression. Indeed, ectopic ZEB1 expression promoted 393P tumor growth and metastasis (Supplemental Figure 3, A and B), and Itga1 depletion abrogated ZEB1-induced cell proliferation on Col1-coated wells without affecting cell proliferation on plastic (Figure 3, A and B). When coinjected with Col1, 344SQ cells grew into larger tumors and produced more lung metastasis than did cells coinjected with Matrigel or phosphate-buffered saline, and this effect was abrogated by Itga1 depletion (Figure 3, C–E). In line with these observations, Itga1-deficient tumor cells exhibited decreased migratory and invasive activities when seeded in collagen-coated Boyden chambers or in collagen gels (Figure 3, F and G, and Supplemental Figure 3, C–F). Itga1 reconstitution in Itga1-deficient cells restored cell invasion in Col1 gels (Figure 3F and Supplemental Figure 3G). When seeded on Col1-coated plates, Itga1-deficient cells were less migratory (Figure 3H) and formed smaller and more numerous focal adhesions that were similar in number and size to those induced by Itgb1-deficient cells (Figure 3I), suggesting that Itga1 is essential for the maturation of focal adhesions on Col1.

### ZEB1 epigenetically increases the expression of Itga1.

**Figure 4:**
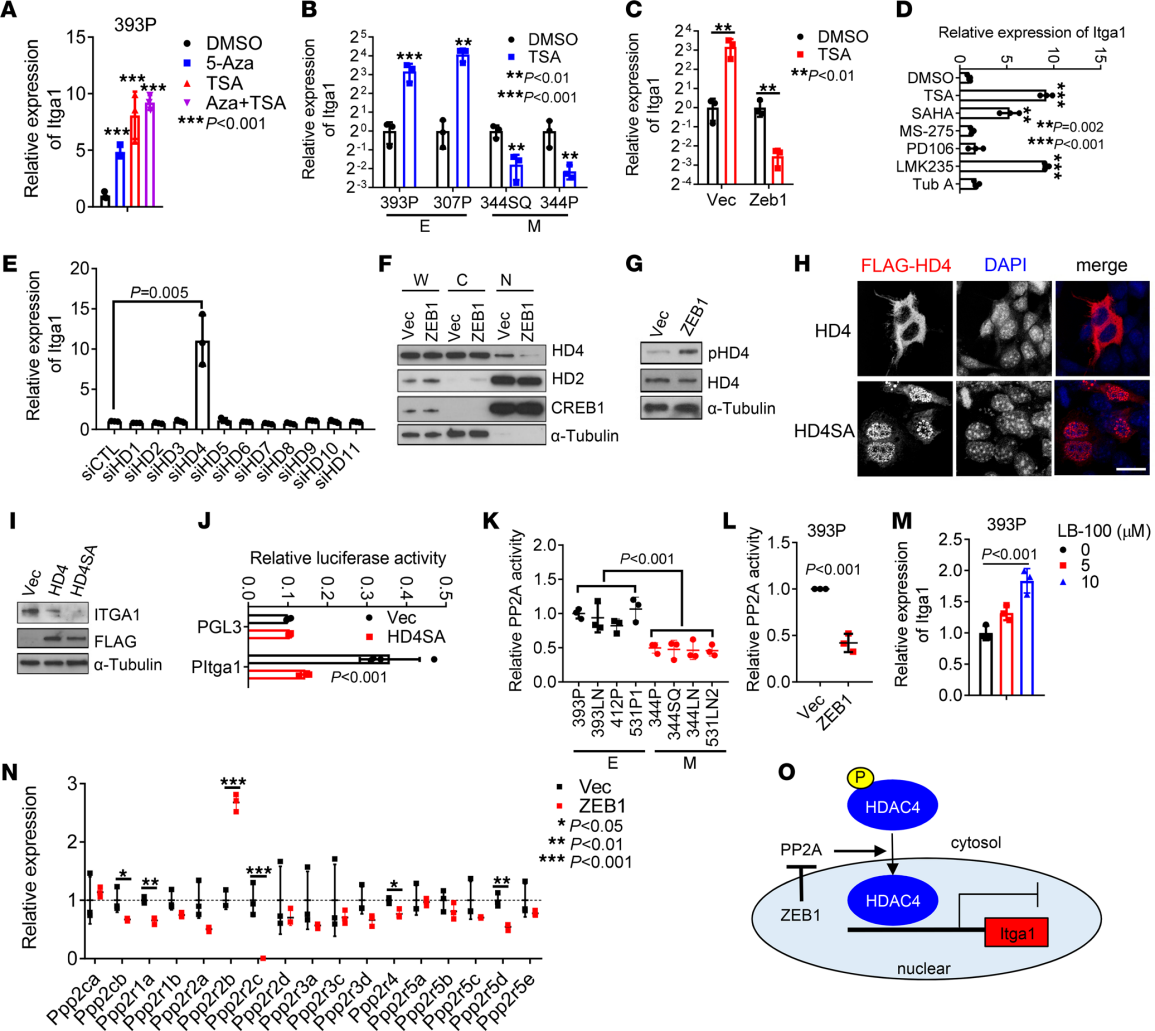
ZEB1 derepresses Itga1 transcription by regulating HDAC4 nuclear translocation. (**A**) Quantitative reverse transcription PCR analysis of Itga1 in 393P cells treated with 5-azacytidine-2′-deoxycytidine (5-Aza) or trichostatin A (TSA) alone or in combination. ***P < 0.001. (**B** and **C**) Quantitative reverse transcription PCR analysis of Itga1 in KP cells treated with or without TSA. E, epithelial cells; M, mesenchymal cells. **P < 0.01; ***P < 0.001. (**D** and **E**) Quantitative reverse transcription PCR analysis of Itga1 in 393P cells treated with indicated histone deacetylase (HDAC) inhibitors (**D**) or transfected with indicated siRNAs (**E**). **P < 0.002; ***P < 0.001. (**F**) Western blot analysis of HDAC4 (HD4), HDAC2 (HD2), CREB, and α-tubulin in indicated cell fractions. W, whole-cell lysate; C, cytoplasmic fraction; N, nuclear fraction. (**G**) Western blot analysis of phospho-HDAC4 (Ser246) (pHD4) and total HD4. (**H**) Confocal micrographs of Flag-tagged wild-type HD4 or mutant HD4 (S246/467/632A) (HD4SA) in 344SQ cells. Scale bar: 20 μm. (**I**) Western blot analysis of Itga1 and ectopic HD4 (FLAG) in 344SQ transfectants. (**J**) Luciferase reporter assay in 393P cells transfected with Itga1 promoter reporter (PItga1) and HD4SA expression vector or control vector (Vec). (**K** and **L**) PP2A activity in 393P_Vec and 393P_ZEB1 cells (**K**) and KP cell panel (**L**). (**M**) Quantitative reverse transcription PCR analysis of Itga1 in 393P cells treated with PP2A inhibitor LB-100 for 1 day. (**N**) Relative expression levels of PP2A subunits in 393P_Vec and 393P_ZEB1 cells quantified by RNA sequencing. *P < 0.05; **P < 0.01; ***P < 0.001. (**O**) Schema showing that ZEB1 depresses Itga1 expression by modulating HDAC4 nuclear accumulation through suppression of PP2A activity. Data are shown as the mean ± SEM from a single experiment incorporating biological replicate samples (*n* = 3, unless otherwise indicated) and are representative of at least 2 independent experiments. Two-tailed Student’s *t* test for 2-group comparisons; 1-way ANOVA test for multiple comparisons.

The above findings warranted further studies to elucidate the way that ZEB1 upregulates Itga1. Ectopic ZEB1 expression in 393P cells upregulated Itga1 levels by more than 100-fold, which led us to suspect that Itga1 is epigenetically silenced in epithelial cells. In line with this possibility, treating epithelial 393P cells or human lung cancer HCC827 cells with the DNA methyltransferase inhibitor 5-azacytidine or the histone deacetylase (HDAC) inhibitor trichostatin A (TSA) significantly upregulated Itga1 levels (Figure 4A and Supplemental Figure 4A). We have previously shown that Itga1 promoter activity is regulated by methylation (25), but the way in which HDACs control the expression of Itga1 is unexplored. We found that TSA treatment increased Itga1 expression levels in epithelial KP cells but decreased Itga1 levels in mesenchymal KP cells or epithelial KP cells that have ectopic ZEB1 expression (Figure 4, B and C). To determine which HDAC is responsible for Itga1 silencing in epithelial KP cells, we treated 393P cells with a panel of HDAC inhibitors and found that pan-HDAC inhibitors and class IIA HDAC (HDAC4, -5, -7, and -9) inhibitors strongly induced the expression of Itga1 (Figure 4D). Furthermore, depletion of HDAC4, but not the other HDACs, increased the expression of Itga1 in epithelial KP cells (Figure 4E and Supplemental Figure 4, B and C), suggesting that HDAC4 is responsible for Itga1 silencing in epithelial KP cells. Class IIA HDACs shuttle between the nucleus and cytoplasm in a phosphorylation-dependent manner (26). HDAC4 protein levels were not downregulated in mesenchymal KP cells or decreased by ectopic ZEB1 expression (Supplemental Figure 4, D and E), whereas ectopic ZEB1 expression reduced HDAC4 nuclear localization and increased HDAC4 phosphorylation (Figure 4, F and G, and Supplemental Figure 4F). Ectopic expression of a phosphorylation-deficient mutant HDAC4 localized primarily in the nucleus and strongly suppressed Itga1 expression levels (Figure 4, H–J), suggesting that ZEB1 upregulates Itga1 by inhibiting HDAC4 nuclear localization.

The tumor suppressor protein phosphatase 2A (PP2A) promotes HDAC4 accumulation in the nucleus via dephosphorylation of Ser298 (27). PP2A activity was lower in mesenchymal cells than in epithelial KP cells and in ectopic ZEB1-expressing cells than in control 393P cells (Figure 4, K and L). PP2A inhibition increased Itga1 expression in 393P cells (Figure 4M). By analyzing the RNA-sequencing data from 393P_Vec and 393P_ZEB1 cells (25), we found that several PP2A subunits were downregulated in ZEB1 cells, including the catalytic subunit Ppp2cb and regulatory subunits Ppp2r1a, Ppp2r2c, Ppp2r4, and Ppp2r5d (Figure 4N), suggesting that ZEB1 may modulate PP2A activity by suppressing the expression of PP2A subunits (Figure 4O).

**Figure 5:**
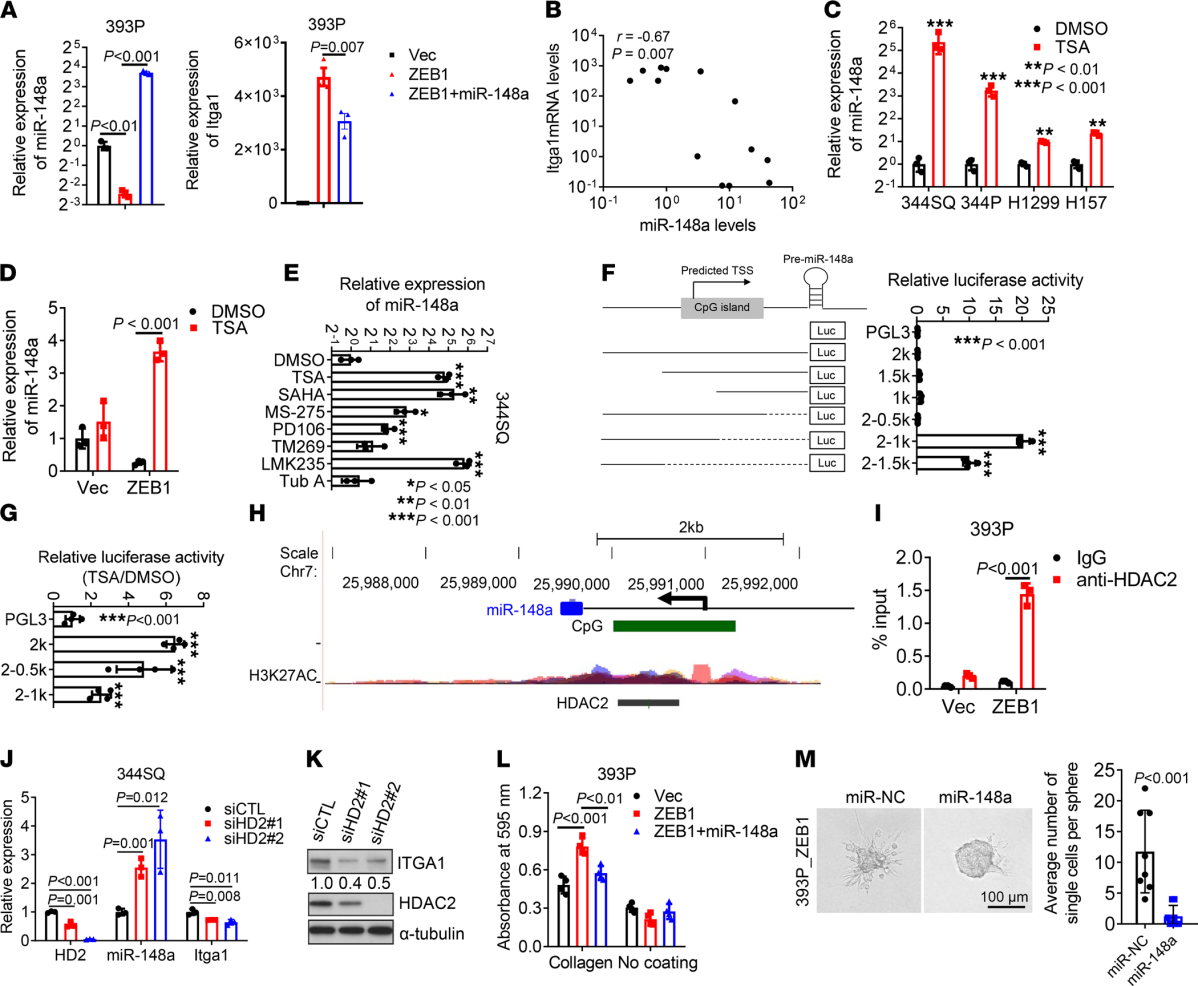
ZEB1 increases Itga1 by epigenetically silencing miR-148a. (**A**) Quantitative reverse transcription PCR analysis of miR-148a (left) and Itga1 (right) in 393P transfectants. (**B**) Pearson correlation between miR-148a and Itga1 expression levels in KP cell lines. (**C** and **D**) Quantitative reverse transcription PCR analysis of miR-148a levels in 344SQ, 344P, H1299, and H157 cells treated with trichostatin A (TSA) or DMSO (**C**) and in 393P_Vec and 393P_ZEB1 cells treated with TSA or DMSO (**D**). **P < 0.01; ***P < 0.001. (**E**) Quantitative reverse transcription PCR analysis of miR-148a in 344SQ cells treated with indicated HDAC inhibitors. *P < 0.05; **P < 0.01; ***P < 0.001. (**F**) Luciferase reporter assays in 393P cells transfected with miR-148a promoter reporters. *n* = 4. (**G**) Luciferase reporter assays in 393P cells transfected with miR-148a promoter reporters and treated with TSA or DMSO. *n* = 4. ***P < 0.001. (**H**) Snapshot of the UCSC genome browser showing CpG island and H3K27AC peaks and the HDAC2-binding site in the miR-148a promoter region. (**I**) Chromatin immunoprecipitation followed by quantitative reverse transcription PCR analysis of HDAC2 occupation on miR-148a promoters. (**J**) Quantitative reverse transcription PCR analysis of HDAC2 (HD2), miR-148a, and Itga1 expression in 344SQ transfectants. (**K**) Western blot analysis of Itga1 and HD2 in 344SQ transfectants. (**L**) Cell adhesion to Col1 or plastic after 1 hour. (**M**) Bright-field micrograph of spheroids formed by 393P_ZEB1 and H1299 transfectants in collagen. Scale bar: 100 μm. Invading single cells per sphere were quantified. Data are shown as the mean ± SEM from a single experiment incorporating biological replicate samples (*n* = 3, unless otherwise indicated) and are representative of at least 2 independent experiments. Two-tailed Student’s *t* test for 2-group comparisons; 1-way ANOVA test for multiple comparisons.

Next, we explored how HDAC inhibition repressed the expression of Itga1 in mesenchymal cancer cells. We have previously shown that Itga1 is a target of miR-148a (25). Ectopic expression of miR-148a inhibited ZEB1-induced Itga1 expression (Figure 5A), and the expression levels of miR-148a and Itga1 were negatively correlated in KP cell lines (Figure 5B). TSA treatment induced the expression of miR-148a in mesenchymal KP cells and human lung cancer cells (Figure 5, C and D). Pan-HDAC inhibitors and class I-HDAC inhibitors significantly induced the expression of miR-148a in 344SQ cells (Figure 5E). Promoter truncation analysis identified a silencer (from –1000 to –500) in the proximal promoter region of the *miR-148a* gene (Figure 5F); this silencer was responsible for HDAC inhibition and was potentially bound by HDAC2 (Figure 5, G and H). Chromatin immunoprecipitation assays confirmed that HDAC2 occupied the miR-148a promoter in a ZEB1-dependent manner (Figure 5I). Depletion of HDAC2 increased the expression of miR-148a and decreased the expression of Itga1 (Figure 5, J and K). miR-148a overexpression in 393P_ZEB1 cells reduced cell adherence to Col1 and cell migration in 3D collagen, which phenocopied the effects of Itga1 knockdown (Figure 5, L and M). Thus, ZEB1 activates a transcriptional program that controls lung adenocarcinoma cell adherence to Col1.

### ZEB1 promotes Col1 secretion.

**Figure 6:**
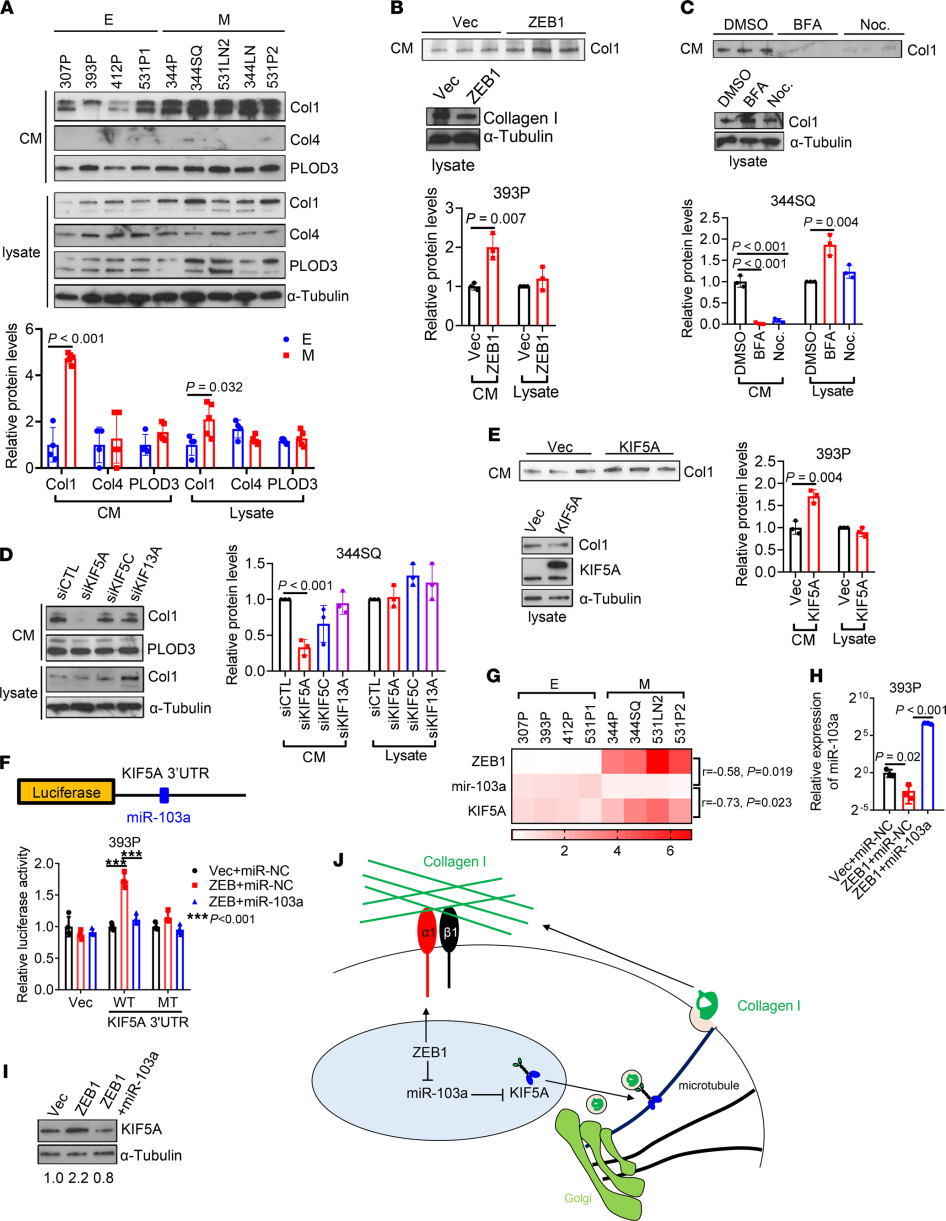
ZEB1 accelerates Col1 secretion. (**A**) Western blot analysis of Col1, Col4, and PLOD3 in conditioned medium (CM) and cell lysates from KP cell lines. Protein levels were quantified. α-Tubulin was used as a loading control. E, epithelial; M, mesenchymal. (**B** and **C**) Western blot analysis of Col1 levels in CM and cell lysates from 393P_Vec and 393P_ZEB1 cells (**B**) and 344SQ cells treated with brefeldin A (BFA), nocodazole (Noc.), or vehicle (DMSO; **C**). (**D**) Western blot analysis of Col1 and PLOD3 in CM and cell lysates from 344SQ transfectants. α-Tubulin was used as a loading control. Col1 protein levels were quantified. (**E**) Western blot analysis of Col1 and KIF5A in CM and cell lysates from 393P transfectants. α-Tubulin was used as a loading control. Col1 protein levels were quantified. (**F**) Schema showing luciferase reporter containing KIF5A 3′-UTR that has a miR-103a–binding site. Luciferase reporter assays in 393P cells transfected with reporters containing wild-type KIF5A 3′-UTR or 3′-UTR with mutant miR-34a binding site (MT), ZEB1 expression vector or control vector, and miR-103a mimics or control miRNA mimics (miR-NC). (**G**) Heatmap showing the expression levels of ZEB1, miR-103a, and KIF5A in KP cell lines. *r* and *P* values were calculated by Pearson correlation. (**H**) Quantitative reverse transcription PCR analysis of miR-103a in 393P transfectants. (**I**) Western blot analysis of KIF5A in 393P transfectants. Relative KIF5A protein levels were quantified. (**J**) Schema showing that ZEB1 drives Col1 secretion by upregulating KIF5A and enhances cell adhesion to Col1 by increasing Itga1. Data are shown as the mean ± SEM from a single experiment incorporating biological replicate samples (*n* = 3, unless otherwise indicated) and are representative of at least 2 independent experiments. Two-tailed Student’s *t* test for 2-group comparisons; 1-way ANOVA test for multiple comparisons.

Although fibroblasts are generally considered to be the primary source of intratumoral collagen (28), tumor cells are emerging as an important source of collagen deposition (29). To determine whether EMT regulates collagen secretion, we compared collagen secretion in epithelial and mesenchymal KP cells and found that mesenchymal KP cells secreted about 4 times more Col1, but not collagen IV, and that intracellular Col1 levels were similar (Figure 6A), suggesting that higher secretion was not a consequence of increased intracellular Col1 levels. Similarly, ectopic ZEB1 expression enhanced Col1 secretion but not intracellular Col1 levels (Figure 6B).

To assess how ZEB1 enhances Col1 secretion, we initially treated cells with the ARF1 inhibitor brefeldin A or the microtubule polymerization inhibitor nocodazole and found that both treatments inhibited Col1 secretion (Figure 6C), suggesting that Col1 secretion is Golgi and microtubule dependent; this led us to speculate that ZEB1 promotes the biogenesis of Col1-containing vesicles. However, analysis of vesicle fractions from 393P_Vec and 393P_ZEB1 cells demonstrated similar levels of Col1 (Supplemental Figure 5, A and B), arguing against this possibility. Based on evidence that a kinesin protein, KIF5A, is necessary for Col1 vesicle transportation in myofibroblasts (30), we examined RNA-sequencing data from 393P_Vec and 393P_ZEB1 cells and found that 3 kinesin genes (KIF5A, KIF5C, and KIF13A) were more highly expressed in 393P_ZEB1 cells (GSE102337; ref. 25) (Supplemental Figure 5C). Depletion of KIF5A, but not the other 2 kinesins, reduced the secretion of Col1 in 344SQ cells (Figure 6D and Supplemental Figure 5, D and E). Conversely, ectopic expression of KIF5A promoted Col1 secretion in 393P cells (Figure 6E). By analyzing the 3′-UTR of KIF5A, we identified a binding site for miR-103a-3p, a ZEB1-silenced miRNA (31). Luciferase reporter assays confirmed that KIF5A is a direct target of miR-103a-3p (Figure 6F). Moreover, KIF5A mRNA levels were negatively correlated with miR-103a and positively correlated with ZEB1 in KP cells and in The Cancer Genome Atlas lung adenocarcinoma cohort (Figure 6G and Supplemental Figure 5, F and G). Ectopic expression of miR-103a-3p reduced KIF5A and collagen secretion in 393P_ZEB1 cells (Figure 6, H and I, and Supplemental Figure 5H). We conclude that ZEB1 accelerates the transport and secretion of Col1-containing vesicles by increasing KIF5A levels.

## Discussion

In one working hypothesis, metastasis requires that cancer cells first undergo EMT to gain migratory and invasive activity within the primary tumor and then switch back to an epithelial state to facilitate the colonization and outgrowth of disseminated tumor cells in the metastatic niche (2). The “seed and soil” hypothesis of metastasis offers a different perspective, in which cancer cells disseminate within the primary tumor by remodeling collagen and other matrix molecules to create invasive tracks, and the disseminated tumor cells then seed distant organs that contain a soil made up of specific matrix molecules that support the colonization and outgrowth of metastatic tumor cells (1, 16). Whether the biological processes described by these parallel viewpoints of the metastatic cascade are mechanistically linked has not been addressed. In the present study, we have shown that ZEB1 promotes tumor cell dissemination by enhancing tumor cell adherence to collagen fibers and remodels the tumor microenvironment by increasing collagen deposition (Figure 6J).

In contrast to a growing body of evidence that Col1 drives cancer progression (10, 24, 32, 33), Col1 can also play a tumor-restricting role. For example, in human pancreatic ductal adenocarcinoma (PDAC), infiltrating fibroblasts generate a dense stromal infiltrate (34), and genetic approaches to eliminate Col1 from myofibroblasts in PDAC mouse models accelerate tumor progression owing to the emergence of a CXCL5-expressing tumor cell population that suppresses antitumor immunity by recruiting myeloid cell–derived suppressor cells (24). In stark contrast to this evidence that Col1 promotes antitumor immunity, Col1 can inactivate CD8-positive T cells by binding to the LAIR-1 collagen receptor (33). Providing additional layers of complexity, cancer cells express multiple collagen receptors that may play opposing roles in tumor progression (17, 35, 36), and ITGA1 mRNA encodes not only a collagen receptor but also a miRNA sponge that functions in a competing endogenous RNA network that influences diverse signaling pathways (25). Furthermore, we show that EMT state governs ITGA1 levels; ITGA1 levels are low in epithelial cells owing to high nuclear HDAC4 activity and are derepressed in mesenchymal cells owing to high ZEB1 levels that inhibit HDAC4 nuclear translocation and silence an ITGA1-targeting miRNA. Thus, the way in which Col1 influences tumorigenesis is highly contextual, owing to the types of collagen receptors expressed on tumor cells, EMT and other supervisory processes in tumor cells, and the types of fibroblasts and immune cells in the tumor microenvironment.

In this study, we found that ITGA1 is essential for lung cancer cells to adhere to Col1 and undergo metastatic dissemination and that ITGA1 and ZEB1 levels are tightly correlated in multiple human cancer types, suggesting that ITGA1-mediated adherence to collagen may be a general effector of EMT in multiple cancer types. Therapeutic strategies designed to inhibit ITGA1 activity have been developed, including small-molecule α_1_β_1_ inhibitors, but these approaches have failed to demonstrate efficacy (37, 38). Given the dichotomous role that Col1 plays in tumorigenesis, these negative outcomes may have resulted in part from including patients in whom Col1 does not drive tumor progression. RNA-sequencing approaches can identify tumors with EMT-related gene expression signatures (39, 40). Based on the findings reported here, revisiting integrin targeting in patient populations selected on the basis of EMT-related expression signatures might be warranted. Based on evidence reported here that HDAC2 derepresses ITGA1 in mesenchymal lung adenocarcinoma, such approaches could include HDAC2 inhibitors that are under clinical development (41). Given that EMT is a key driver of acquired resistance to kinase inhibitors in lung adenocarcinoma (39), ITGA1 targeting strategies could address an immediate clinical need.

## Methods

### Antibodies and plasmid constructs.

Antibodies against Itga1 (Santa Cruz, sc-271034), β-actin (Cell Signaling Technology, 4970), Itgb1 (Cell Signaling Technology 4706 for Western blot; GeneTex GTX128839 for coimmunoprecipitation), phospho-paxillin (Tyr118) (Cell Signaling Technology, 2541), HDAC2 (Cell Signaling Technology, 57156), HDAC4 (Cell Signaling Technology, 15164), phospho-HDAC4 (Ser246) (Cell Signaling Technology, 3443), HDAC5 (Cell Signaling Technology, 98329), HDAC6 (Cell Signaling Technology, 7558), CREB1 (Cell Signaling Technology, 9197), FLAG tag (MilliporeSigma, F1804), α-tubulin (MilliporeSigma, T9026), ZEB1 (Santa Cruz, sc-25388), KIF5A (Proteintech, 21186-1-AP), Collagen I (Abcam, ab34710), PLOD3 (Proteintech, 60058-1-Ig), and Collagen IV (Proteintech, 55131-1-AP) were used. Brefeldin A (MilliporeSigma, B5936), nocodazole (MilliporeSigma, 31430-18-9), 5-azacytidine-2′-deoxycytidine (MilliporeSigma, A3656), TSA (MilliporeSigma, T1952), SAHA (MedchemExpress, HY- 10221), MS-275 (MedchemExpress, HY-12163), PD106 (MedchemExpress, HY-19348), LMK-235 (MedchemExpress, HY-18998), and tubacin A (MedchemExpress, HY-13428) were used. Gene-specific shRNAs and siRNAs and miRNA mimics were purchased from MilliporeSigma: mouse shItga1 no. 1 to no. 5 (TRCN0000254183, TRCN0000265481, TRCN0000254182, TRCN0000254180, and TRCN0000254181), mouse siItga1 (SASI_Mm01_00102775 and SASI_Mm02_00288356), mouse siHDAC1 (SASI_Mm02_00313417), siHDAC2 (SASI_Mm01_00100698), siHDAC3 (SASI_Mm02_00318546), siHDAC4 (SASI_Mm01_00195677, SASI_Mm01_00195678, and SASI_Mm01_00195679), siHDAC5 (SASI_Mm02_00318547), siHDAC6 (SASI_Mm02_00311178), siHDAC7 (SASI_Mm01_00133671), siHDAC8 (SASI_Mm01_00107278), siHDAC9 (SASI_Mm01_00144986), siHDAC10 (SASI_Mm02_00351385), siHDAC11 (SASI_Mm01_00123735), siKIF5A (SASI_Mm01_00175495, SASI_Mm01_00175496, SASI_Mm01_00175497), human siITGA1 no. 1 and no. 2 (SASI_Hs01_00067020 and SASI_Hs01_00067021), and miRNA mimics (HMI0237 and HMI0270). pcDNA3-HDAC4-FLAG and pcDNA3-HDAC4.3SA-FLAG (Addgene plasmid 30485 and 30486) were gifts from Tso-Pang Yao (Duke University, Durham, North Carolina, USA) (42). KIF5A-pEGFP-C1 expression vector (Addgene plasmid 31607) was a gift from Anthony Brown (The Ohio State University, Columbus, Ohio, USA) (43). Itga1 coding sequence was amplified from the cDNA from 344SQ cells and cloned into pLVX-puro vector (Clontech). Itga1 promoter fragments were amplified from the genomic DNA of 344SQ cells and subcloned into PGL3-Basic vector (Promega). PCR primers are listed in Supplemental Table 2.

### Cell culture and functional assays.

Murine lung cancer cell lines (713P, 307P, 344LN, 393LN, 393P, 412P, 344SQ, 344P, 531LN1, 531LN2, 531P1, and 531P2) were derived from KP mice as described previously (20). β1-depleted 344SQ cells were described previously (44). Human lung cancer cells (HCC827, H157, and H1299) were purchased from the ATCC and cultured in RPMI 1640 (Corning) supplemented with 10% fetal bovine serum (Gibco). Cells were transfected using jetPRIME Versatile DNA/siRNA transfection reagent (Polyplus). Stable cell transfectants were selected using puromycin (InvivoGene) or G418 (Corning). When indicated, 2 μM 5-azacytidine-2′-deoxycytidine, 1 μM TSA, 5 μM SAHA, 2 μM MS-275, 5 μM PD106, 2 μM LMK235, or 2 μM tubacin A was added into culture medium. For proliferation assays, 2000 cells were plated in 96-well plates, and the cellular proliferation was assessed using the WST-1 reagent (Roche) according to the manufacturer’s instructions. For adhesion assays, 1 × 10^5^ cells were seeded on 24-well plates coated with or without Col1 (50 μg/ml) and incubated for 1 to 2 hours. After the attached cells were washed twice with phosphate-buffered saline, they were stained with 0.1% crystal violet and optical density was measured at 595 nm. For migration and invasion assays, 2 × 10^4^ cells were cultured in the upper wells of Transwell and Matrigel chambers, respectively (BD Biosciences) and allowed to migrate toward 10% fetal bovine serum in the bottom wells. After 8–10 hours of incubation, migrating or invading cells were stained with 0.1% crystal violet, photographed, and counted. 3D collagen cell migration assays were performed as previously described (45). Cell spheroids were imaged under a bright-field microscope 12 or 24 hours after seeding and single migratory cells were counted. For single-cell tracing experiments, live-cell microscopy was carried out for 18–24 hours on 0.5 mg/ml Col1-coated microwell plates under a Nikon Eclipse Ti live cell imaging microscope (Nikon Corporation) using differential interference contrast imaging mode with a 20×/0.75 NA dry objective. Centroids of individual cells were computed for tracking analysis with Imaris (Bitplane). A total of 50–100 cells were analyzed per condition.

### Quantitative reverse transcription PCR.

Total RNA was isolated from cells using TRIzol (Life Technologies) and subjected to reverse transcription using the qScript cDNA superMix (Quanta Biosciences). mRNA levels were determined using SYBR Green Real-Time PCR Master Mixes (Bimake) and normalized on the basis of ribosomal protein L32 (Rpl32) mRNA. miRNA levels were quantified using stem-loop reverse transcription PCR assays and normalized to U6 snRNA levels. The primer sequences for quantitative PCR are listed in Supplemental Table 2.

### Luciferase reporter assays.

For the promoter assays, cells were seeded on 48-well plates (5 × 10^4^ cells/well) and transiently transfected 24 hours later with 200 ng luciferase reporter plasmids, 50 ng pRL-TK control vector, and 250 ng Zeb1 expression vector or empty vector. For the 3′-UTR assays, 3′-UTR reporters (10 ng), pGL3-control (50 ng), and miRNA mimics (10 nM) or miRNA expression vectors (250 ng) were cotransfected into cells. After 24 hours, luciferase activity was measured using the Dual-Luciferase Reporter Assay System (Promega).

### Chromatin immunoprecipitation.

As described previously (21), cell lysates were subjected to cross-linking, followed by sonication with a Cole-Parmer GEX-130 ultrasonic processor using 50% power (pulse on for 10 seconds, pulse off for 10 seconds; 20 cycles) and immunoprecipitation with anti-RNA polymerase II or anti-rabbit IgG (Santa Cruz). DNA was eluted and purified using the MinElute Reaction Cleanup kit (Qiagen) and subjected to quantitative PCR. PCR primers are listed in Supplemental Table 2.

### Western blot and coimmunoprecipitation.

For Western blot analysis, protein lysates were separated on a 4%–20% Bis-Tris gel and transferred to polyvinylidene difluoride membranes using Trans-Blot Turbo Transfer System (Bio-Rad). The membranes were blocked in 5% milk and probed with primary antibodies following a standard protocol. Coimmunoprecipitation of Itga1 and Itgb1 was performed as previously described (46). Briefly, 344SQ cell lysates were immunoprecipitated with anti-Itgb1 or IgG control, and the immunoprecipitates were subjected to Western blot analysis using anti-Itgb1 or -Itga1 antibody. See complete unedited blots in the supplemental material.

### Immunofluorescence.

Analysis of the number and size of focal adhesions was performed using immunofluorescence staining for phospho-paxillin, followed by confocal microscopy, image processing, and analysis as described previously (45). Analysis of Flag-tagged HDAC4 localization was performed by staining for Flag.

### Isolation of Golgi and vesicle fractions.

As previously described (47), Golgi and vesicle fractions were enriched using the Minute Golgi Apparatus Enrichment Kit (GO-037, Invent Biotechnologies) according to the manufacturer’s instructions.

### PP2A activity assay.

PP2A activity was assessed using the PP2A Immunoprecipitation Phosphatase Assay Kit (MilliporeSigma, 17-313) according to the manufacturer’s instructions. Briefly, 2 × 10^6^ cells were lysated in RIPA buffer, and the whole-cell extractions were incubated with 2 μg anti-PP2A antibody for 2 hours and then with 30 μl protein A agarose beads for 2 hours at 4°C. After washing with Tris-buffered saline (MilliporeSigma), the immunoprecipitates were incubated with threonine phosphopeptide (K-R-pT-I-R-R) for 10 minutes at 30°C in a shaking incubator. The resulting phosphate was quantified with Malachite Green Phosphate Detection Solution (MilliporeSigma).

### Conditioned medium assays.

As previously described (47), conditioned medium samples were isolated, filtered through a 0.45 μm filter, and concentrated using Amicon Ultra Centrifugal Filters (MilliporeSigma). The resulting conditioned medium samples were mixed with 1× SDS loading buffer, denatured at 98°C for 5 minutes, and analyzed by Western blot.

### Animal husbandry.

Immunocompetent 129/Sv mice syngeneic with were bred in-house. Six- to 8-week-old male mice were either injected subcutaneously in the right flank with 10^6^ tumor cells (8–10 mice per group) and necropsied after 5 weeks or injected intravenously with 10^5^ tumor cells (5–8 mice per group) and necropsied after 1 or 5 days. Primary tumors were weighed, and lung metastases on the pleural surfaces were counted. Mice received standard care and were euthanized according to the standards set forth by the Institutional Animal Care and Use Committee of The University of Texas MD Anderson Cancer Center.

### Statistics.

Unless stated otherwise, the results shown are representative of replicated experiments; data represent the mean ± SD from triplicate samples or randomly chosen cells within a field. Statistical evaluations were carried out with Prism 6 (GraphPad). Unpaired 2-tailed Student’s *t* tests were used to compare the mean values of 2 groups. One-way ANOVA with the Dunnett’s test was used for comparing multiple treatments with a control. *P* < 0.05 was considered statistically significant. When performing the correlation analysis and comparing the mRNA levels of collagen receptors with EMT scores in human lung cancers, the EMT score was calculated as previously described (22).

### Study approval.

All mouse studies were approved by the Institutional Animal Care and Use Committee of The University of Texas MD Anderson Cancer Center (00000957-RN02).

## Author contributions

JMK and XT designed the research; XT, PB, and XL performed the research; JY, SL, YHA, and CJC contributed new reagents/analytic tools; XT, PB, and CJC analyzed the data; and JMK and XT wrote the paper.

## Supplementary Material

Supplemental data

Supplemental table 1

Supplemental table 2

## Version 1 12/07/2021

In-Press Preview

## Version 2 01/11/2022

Electronic publication
