# Supplementary material for: Transcriptional control of a collagen deposition and adhesion process that promotes lung adenocarcinoma growth and metastasis
Source: JCI Insight. 2022 Jan 11;7(1):e153948. doi: 10.1172/jci.insight.153948 (PMC8765047; doi:10.1172/jci.insight.153948)
Supplement: Supplemental data [file jciinsight-7-153948-s149.pdf]

Supplementary Figures

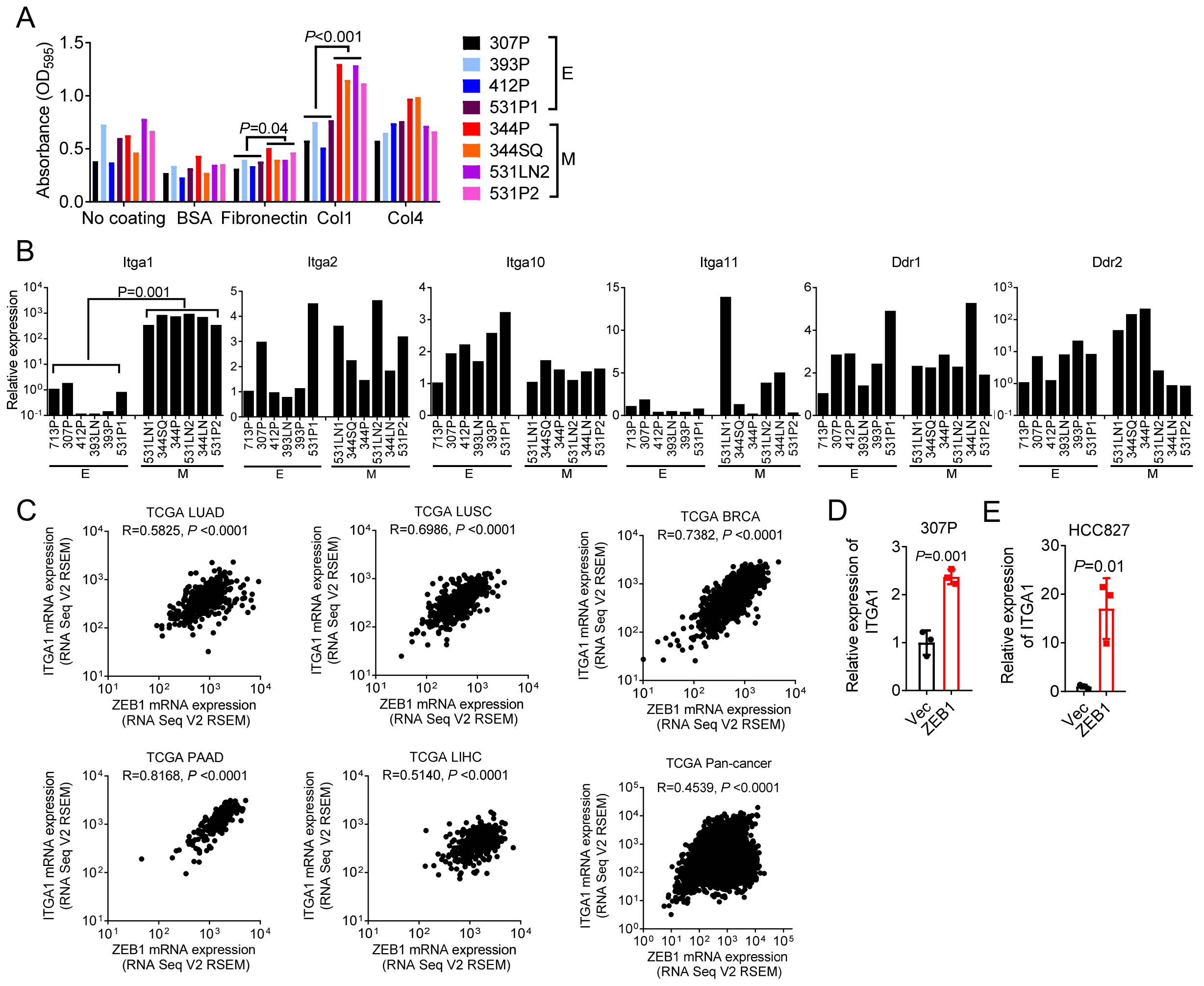

Figure S1. Epithelial-to-mesenchymal transition increases tumor cell adherence to collagen through α1β1 integrin. (A) Cell adherence was quantified 1 hour after the indicated KP cells were seeded on wells coated with Collagen I (Col1), Collagen IV (Col4), fibronectin, bovine serum albumin (BSA), or nothing, and the OD_{595} values were plotted. M indicates mesenchymal; E, epithelial. (B) Quantitative reverse transcription PCR analysis of the mRNA levels of Col1 receptors in KP cell lines. (C) Correlation between ITGA1 and ZEB1 mRNA levels in indicated The Cancer Genome Atlas (TCGA) cancer cohorts. LUAD: lung adenocarcinoma; LUSC: lung squamous cell carcinoma; BRCA: breast cancer; PAAD: pancreatic adenocarcinoma; LIHC: liver hepatocellular carcinoma. R and P values: Pearson correlation. (D and E) Quantitative reverse transcription PCR analysis of the mRNA levels of ITGA1 in 307P_Vec/ZEB1 (C) and HCC827_Vec/ZEB1 (D) cells. Results represent mean ± SD values from a single experiment

incorporating biological replicate samples (n = 3, unless otherwise indicated) and are representative of at least two independent experiments. P values, two-tailed Student's t-test.

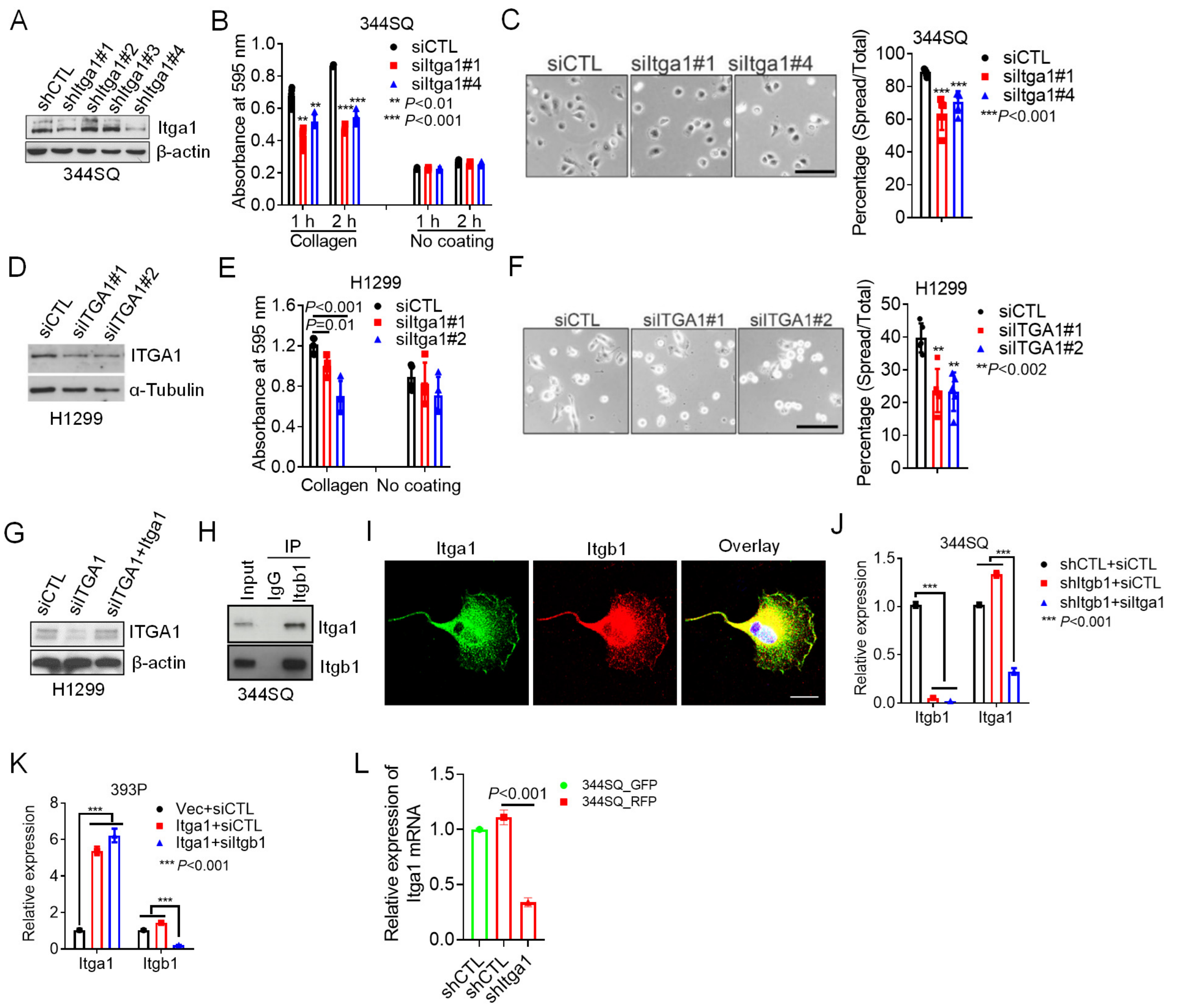

Figure S2. Zeb1 promotes cell adhesion and spreading on collagen by upregulating Itga1. (A and D) Western blot analysis of Itga1 in 344SQ cells (A) and H1299 cells (D) transfected with Itga1 shRNAs (shItga1) or control shRNA (shCTL). (B and E) Cell adhesion to Col1 or plastic at indicated time points. n=4. (C and F) Percentages of spread cells on Col1 at indicated time points were quantified. Scale bar 100 μm. (G) Western blot analysis of ITGA1 in H1299 transfectants. (H) Immunoprecipitation (IP) with Itgb1 antibody or control IgG followed by Western blot analysis in 344SQ cells. (I) Confocal images of 344SQ cells co-stained with Itga1 and Itgb1 antibodies. Scale bar: 10 μm. (J and K) Quantitative reverse transcription PCR analysis of Itga1 and Itgb1 in 344SQ (J) and 393P (K) transfectants. (L) Quantitative reverse transcription PCR analysis of

green or red fluorescent protein (GFP/RFP)-labeled 344SQ transfectants. Results represent mean ± SD values from a single experiment incorporating biological replicate samples (n = 3, unless otherwise indicated) and are representative of at least two independent experiments. P values, two-tailed Student's t-test.

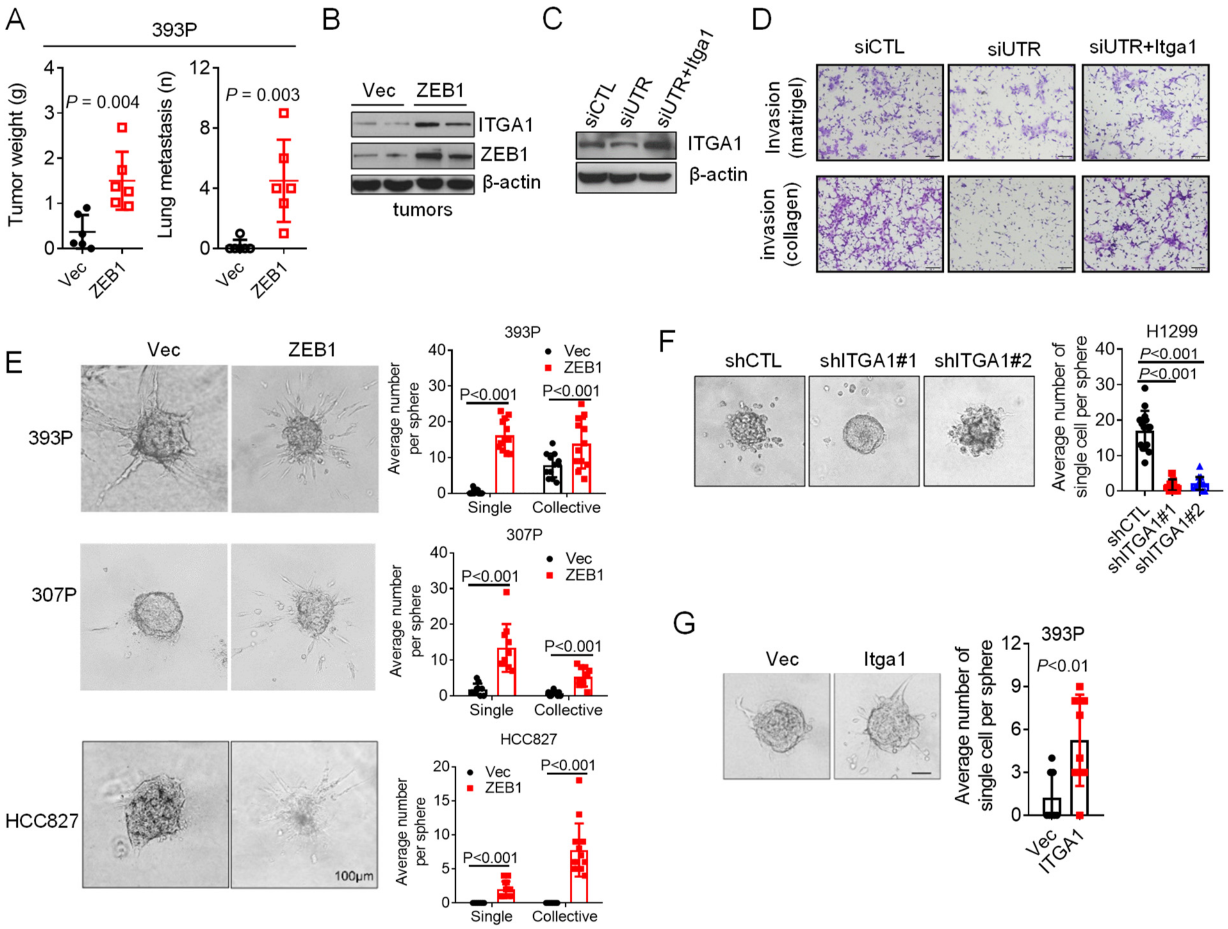

Figure S3. Itga1 mediates Col1-induced cell growth and metastasis. (A) Tumor weight and number of lung metastasis. (B and C) Western blot analysis of Itga1 in tumors (B) and 344SQ transfectants (C). (D) Images of Boyden chamber Matrigel and Col1 invasion assays. (E) Bright field micrograph of spheroids formed by 393P, 307P, and HCC827 transfectants in Col1 showing cells invading singly or collectively. Invading single cells and collective cells per sphere were quantified (graphs). (F and G) Bright field micrograph of spheroids formed by H1299 (F) and 393P (G) transfectants in Col1. Invading single cells per sphere were quantified (graphs). Scale bars: 100 μm. Results represent mean ± SD values from a single experiment incorporating biological

replicate samples (n = 3, unless otherwise indicated) and are representative of at least two independent experiments. P values, two-tailed Student's t-test.

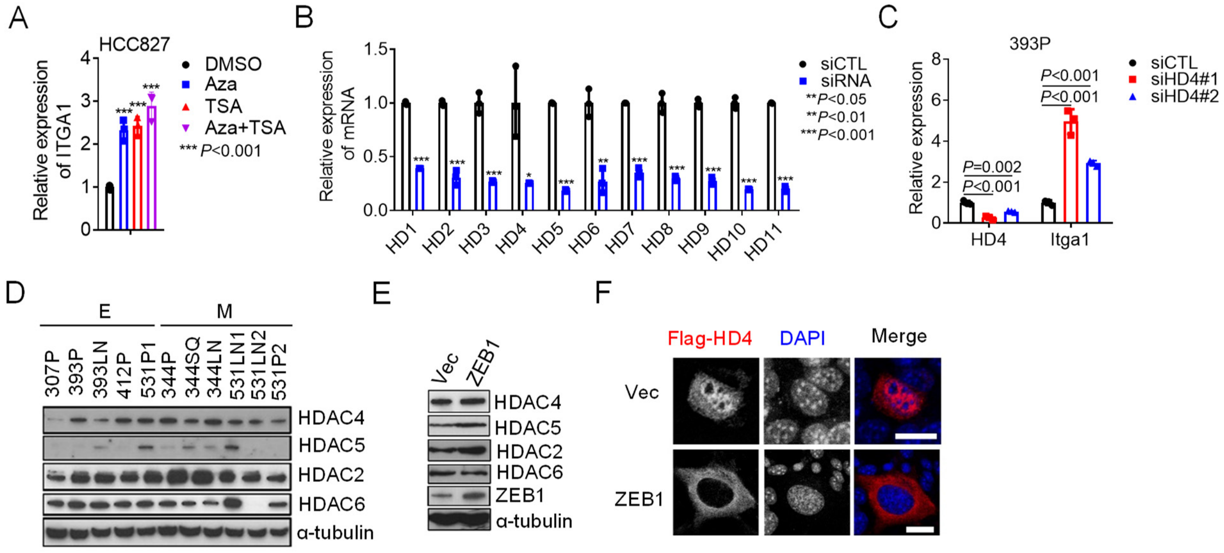

Figure S4. ZEB1 de-represses Itga1 transcription by regulating HDAC4 nuclear translocation. (A) Quantitative reverse transcription PCR analysis of Itga1 in HCC827 cells treated with 5-azacytidine-2′-deoxycytidine (Aza) or trichostatin A (TSA) alone or in combination. (B) Quantitative reverse transcription PCR analysis of HDAC1-11 (HD1-11) in 393P cells transfected with indicated siRNAs. (C) Quantitative reverse transcription PCR analysis of Itga1 and HD4 in 393P transfectants. (D and E) Western blot analysis of HD4, HD5, HD2, and HD6 in KP cell lines (C) and 393P_Vec/ZEB1 cells (D). (F) Confocal micrographs of Flag-tagged HD4 in 393P_Vec/ZEB1 cells. Scale bars: 10 μm. Results represent mean ± SD values from a single experiment incorporating biological replicate samples (n = 3, unless otherwise indicated) and are representative of at least two independent experiments. P values, two-tailed Student's t-test.

Figure S5. ZEB1 accelerates Col1 secretion. (A) Western blot analysis of Col1 in indicated cell fractions. WCE indicates whole cell extracts. (B) Col1 levels in vesicle fractions were quantified. (C) Relative expression levels of Kinesin genes in 393P_Vec/ZEB1 cells quantified by RNA sequencing. (D) Quantitative reverse transcription PCR analysis of KIF5A, KIF5C, and KIF13A in 344SQ transfectants. (E) Western blot analysis of Col1 and KIF5A in conditioned medium (CM) and cell lysates from 344SQ transfectants. Protein levels in the CM were quantified (graph). α-Tubulin was used as a loading control. (F) Correlation between KIF5A and ZEB1 mRNA levels in LUAD samples. r and *P* values were calculated by Pearson correlation. (G) Correlation between miR-103-1/miR-103-2 and ZEB1 in The Cancer Genome Atlas (TCGA) LUAD samples. r and *P* values were calculated by Spearman correlation. (H) Western blot analysis of Col1 in CM from 344SQ transfectants. Col1 protein levels were quantified (graph). Results represent mean ± SD values from a single experiment incorporating biological replicate samples (n = 3, unless otherwise indicated) and are representative of at least two independent experiments. P values, two-tailed Student's t-test.
