# Supplementary material for: Transcriptional control of a collagen deposition and adhesion process that promotes lung adenocarcinoma growth and metastasis
Source: JCI Insight. 2022 Jan 11;7(1):e153948. doi: 10.1172/jci.insight.153948 (PMC8765047; doi:10.1172/jci.insight.153948)
Supplement: Supplemental table 1 [file jciinsight-7-153948-s150.pdf]

Table S1. Cell lines used in this study.

Cell line name	E/M status	mutations
Murine lung cancer cell lines		
307P	E	$Kras^{LA1/+}/Trp53^{R172H\Delta g/+}$
393P	E	$Kras^{LA1/+}/Trp53^{R172H\Delta g/+}$
393LN	E	$Kras^{LA1/+}/Trp53^{R172H\Delta g/+}$
412P	E	$Kras^{LA1/+}/Trp53^{R172H\Delta g/+}$
531P1	E	$Kras^{LA1/+}/Trp53^{R172H\Delta g/+}$
713P	E	$Kras^{LA1/+}/Trp53^{R172H\Delta g/+}$
344P	M	$Kras^{LA1/+}/Trp53^{R172H\Delta g/+}$
344SQ	M	$Kras^{LA1/+}/Trp53^{R172H\Delta g/+}$
344LN	M	$Kras^{LA1/+}/Trp53^{R172H\Delta g/+}$
531LN1	M	$Kras^{LA1/+}/Trp53^{R172H\Delta g/+}$
531LN2	M	$Kras^{LA1/+}/Trp53^{R172H\Delta g/+}$
531P2	M	$Kras^{LA1/+}/Trp53^{R172H\Delta g/+}$
393P Vec	E	$Kras^{LA1/+}/Trp53^{R172H\Delta g/+}$
393P ZEB1	M	$Kras^{LA1/+}/Trp53^{R172H\Delta g/+}$
307P Vec	E	$Kras^{LA1/+}/Trp53^{R172H\Delta g/+}$
307P ZEB1	M	$Kras^{LA1/+}/Trp53^{R172H\Delta g/+}$
Human lung cancer cell lines		
H1299	M	$TP53^{-/-}$
H157	M	$KRAS^{G12R}$ /TP53 (E298*)
HCC827	E	EGFR (exon19del E746–A750)
HCC827_Vec	E	EGFR (exon19del E746–A750)
HCC827 ZEB1	M	EGFR (exon19del E746–A750)
