# Supplementary material for: Transcriptional control of a collagen deposition and adhesion process that promotes lung adenocarcinoma growth and metastasis
Source: JCI Insight. 2022 Jan 11;7(1):e153948. doi: 10.1172/jci.insight.153948 (PMC8765047; doi:10.1172/jci.insight.153948)
Supplement: Supplemental table 2 [file jciinsight-7-153948-s151.pdf]

Table S2: Primers

**qPCR primers**		
**Gene**	**Forward (5′-3′)**	**Reverse (5′-3′)**
Itga1 (mouse)	TGGCTTCTCACCGTTATCCTA	CACACAAGGCATTGATCTCTCT
Itgb1 (mouse)	CTCCAGAAGGTGGCTTTGATGC	GTGAAACCCAGCATCCGTGGAA
ITGA1(human)	CCGAAGAGGTACTTGTTGCAGC	GGCTTCCGTGAATGCCTCCTTT
Rpl32(mouse)	GGAGAAGGTTCAAGGGCCAG	TGCTCCCATAACCGATGTTTG
RPL32 (human)	CCTTGTGAAGCCCAAGATCG	TGCCGGATGAACTTCTTGGT
Zeb1 (mouse)	GCTCAGCCAGGAACCCGCAG	TGGGCACCCTCTGCCACACA
Itgb1 (mouse)	CTACTTCTGCACGATGTGATGAT	TTGGCTGGCAACCCTTCTTT
Itga2 (mouse)	TGTCTGGCGTATAATGTTGGC	CTTGTGGGTTCGTAAGCTGCT
Itga10 (mouse)	CACCAGAGGCCGAATTTGGAT	CCCCAACATGCTGTAAGACAC
Itga11 (mouse)	TGGAGATGTCGCAGACTGGCTT	GAGGAATCACCTTGCCAGCACT
Ddr1 (mouse)	TGCTGCTTCTCATCATCGCGCT	GGACAGAAAGGTGAACCGTCAG
Ddr2 (mouse)	TCATCCTGTGGAGGCAGTTCTG	CTGTTCACTTGGTGATGAGGAGC
Hdac1 (mouse)	TGAAGCCTCACCGAATCCGCAT	TGGTCATCTCCTCAGCATTGGC
Hdac2 (mouse)	GTTTTGTCAGCTCTCCACGGGT	CTTGGCATGATGTAGTCCTCCAG
Hdac3 (mouse)	AACCTCATCGCCTGGCATTGAC	GTAGTCCTCAGAATGGAAGCGG
Hdac4 (mouse)	AGCAGGAGCTGCTCTTCAGACA	ACAGAGGTCTGTGGCTGCCAAA
Hdac5 (mouse)	ACCAGCAGTTCCTGGAGAAGCA	TCCGTCAGCTCCTCTTCTGTCT
Hdac6 (mouse)	TCGCTGTCTCATCCTACCTGCT	GTCAAAGTTGGCACCTTCACGG
Hdac7 (mouse)	CGCCTCAAACTGGATAACGGGA	GCATTGGAGGAATGCAGCTCGT
Hdac8 (mouse)	GTCAGCCAAGAAGGTGATGAGG	ACACTTCCCGTCAATCAGGCAC
Hdac9 (mouse)	CTTGAAGGTGCGGTCCAGGTTA	GCTGCTACTGACCGAGGATTCT
Hdac10 (mouse)	GGCTGGATTTGACTCTGCTATCG	CCTCCAACACAGCACAAATCCG
Hdac11 (mouse)	AAGGCATCTCCAGAGCCACCAT	CAGGGTAGATGTGGCGGTTGTA
Kif5a (mouse)	AGCAGATGCTGGACCAGGAAGA	TTCACCTCGTCCTTCGCAGCAT
**miRNA qPCR RT primers**		
U6-RT	GTCGTATCCAGTGCAGGGTCCGAGGTATTCGCACTGGATACGACAAAATATG	
miR-148a-RT	GTCGTATCCAGTGCAGGGTCCGAGGTATTCGCACTGGATACGACACAAAG	
miR-103a-RT	GTCGTATCCAGTGCAGGGTCCGAGGTATTCGCACTGGATACGACTCATAG	
**miRNA qPCR primers**	**Forward (5′-3′)**	**Reverse (5′-3′)**
U6	GCGCGTCGTGAAGCGTTC	GTGCAGGGTCCGAGGT
miR-148a	CGCGTCAGTGCACTACAGAAC	GTGCAGGGTCCGAGGT
miR-103a	GCAGCATTGTACAGGGCTATG	GTGCAGGGTCCGAGGT
**Clone primers**		
**Primers for promoter cloning (PGL3-Basic)**		
PItga1-2k	CGACGCGTTGATGTTGGAGCAGTAGCTGAG	CCGCTCGAGGACGCCTGGGGACCATTG
**Primers for gene expression (pLVX-puro)**		
Itga1 (mouse)	CCGCTCGAGATGGTCCCCAGGCGTC	GCTCTAGATCATTTCTCCATTTTCTTCTTTAGTG
